# Diagnostic utility of p16 immunocytochemistry for Trichomonas in urine cytology

**DOI:** 10.1186/1742-6413-2-11

**Published:** 2005-06-29

**Authors:** Liron Pantanowitz, Q Jackie Cao, Robert A Goulart, Christopher N Otis

**Affiliations:** 1 Department of Pathology, Baystate Medical Center, Tufts University School of Medicine, Springfield, USA

## Abstract

We present a case in which p16 immunocytochemistry helped establish the diagnosis of *Trichomonas *in urine from a male patient. Based on this finding, we recommend p16 immunocytochemistry as a diagnostic tool for unexpected patients or specimen types in which potential trichomonads are identified following routine cytologic evaluation.

## Article

**Figure 1:**
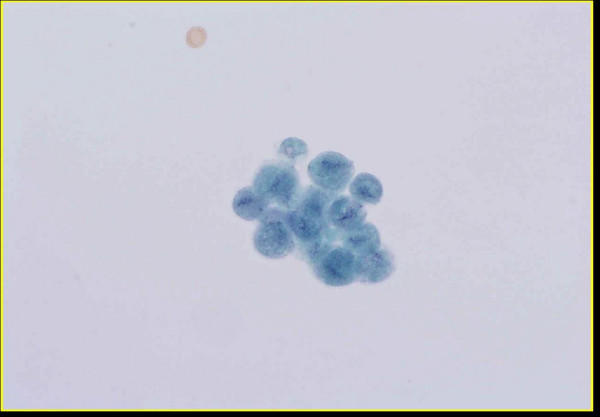
Group of *Trichomonas *organisms present in urine (ThinPrep™, Papanicolaou stain).

We received 50 ml of voided urine from a 84-year-old diabetic male who presented with hematuria. Routine urine cultures yielded no growth after 24 hours. A Papanicolaou-stained ThinPrep™ slide was prepared which revealed benign urothelial cells, neutrophils including "cannonballs" (i.e. neutrophils aggregated around epithelial cells), red blood cells and numerous *Trichomonas *organisms (Figure 1). The diagnosis of *Trichomonas *was based upon the presence of a discernible nucleus and cytoplasmic granules that were identified in several of the trichomonads. A visible nucleus and well-defined cytoplasmic granules at 40x magnification are specified as important morphological features required for a confident diagnosis of *Trichomonas *in liquid-based Pap tests [1]. Although we did not identify flagella in the trichomonads in our case, the finding of flagella, while helpful, is not always required to make a diagnosis of *Trichomonas *[1]. In fact, the morphologic identification of *Trichomonas *on liquid-based Pap tests has been shown to be highly accurate [2]. Nevertheless, exfoliated cells including microorganisms in urine are often degenerated, which makes the identification of *Trichomonas *in these specimens by morphology alone difficult. Therefore, confirmatory testing may be needed. However, traditional methods to detect *Trichomonas *including culture and wet-mount microscopy, as well as molecular studies, may not always be readily available, particularly on fixed samples received in liquid-based vials for cytologic evaluation.

**Figure 2:**
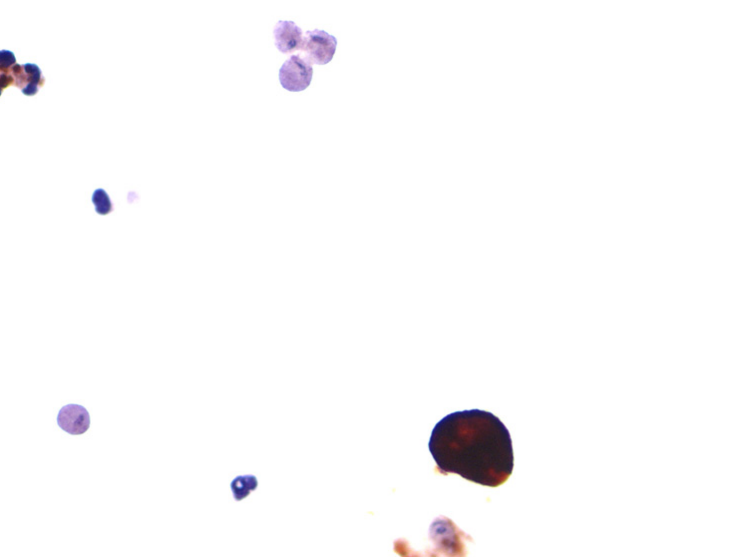
Cytokeratin immunocytochemistry. A single urothelial cell demonstrates strong cytokeratin immunoreactivity whereas surrounding trichomonads are negative. Degenerated inflammatory cells are also present in this field.

**Figure 3:**
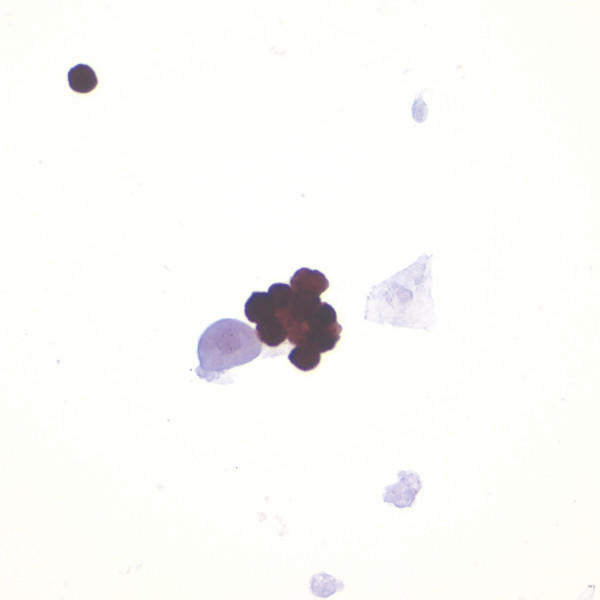
p16 immunocytochemistry. A group of trichomonads demonstrate strong p16 immunoreactivity whereas an adjacent degenerated urothelial cell and squamous cell are negative.

**Figure 4:**
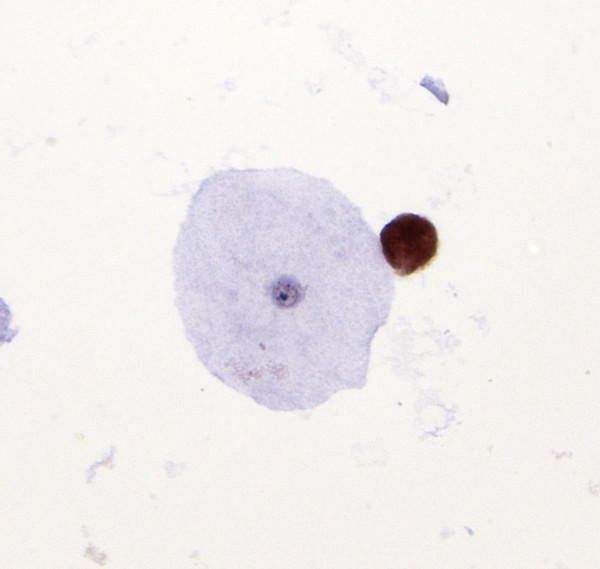
A single trichomonad, adjacent to an unstained exfoliated squamous epithelial cell, is shown to be p16 immunoreactive.

In order to differentiate parasites from degenerated urothelial cells in our case we prepared additional ThinPrep™ slides from the residual specimen to perform immunocytochemistry for cytokeratin (using a cocktail of high- and low-molecular weight keratins) and p16 (using primary purified mouse anti-human p16 antibody, clone G175-405, supplied by BD Biosciences Pharmingen, San Diego, CA, USA). We included p16 based upon recent published data by our group indicating that *Trichomonas vaginalis *organisms in cervicovaginal specimens are immunoreactive for p16 [3]. In all ten of these cervicovaginal specimens *T. vaginalis *were p16 positive and demonstrated strong cytoplasmic staining. Immunocytochemical staining for p16, a proven biomarker for high grade dysplasia associated with Human Papillomavirus (HPV) infection, has been previously applied successfully to cervicovaginal cytology specimens [4]. However, p16 immunoreactivity is not specific for HPV-infected epithelium, as immunoreactivity has previously been documented with inflammatory cells, multinucleated giant cells, bacteria and mucus in cervicovaginal specimens [4-6]. It is unclear if p16 staining of *Trichomonas *organisms reflects specific immunoreactivity (to unknown epitopes) or may be non-specific. In our case, urothelial and squamous cells were strongly immunoreactive for cytokeratin (Figure 2) but were negative for p16, whereas trichomonads demonstrated strong p16 immunoreactivity (Figure 3 and Figure 4) and failed to react with cytokeratin. Appropriate controls were included in this study (data not shown). Following the diagnosis of *Trichomonas*, our patient was treated with a course of metronidazole.

*Trichomonas *infection in male patients may be asymptomatic or associated with non-gonococcal urethritis, prostatitis, and urethral strictures. Protozoa in men may be harbored in the uncircumcised prepuce, urethra, seminal vesicles, prostate gland or bladder. As shown in the present case, it is often difficult to establish the diagnosis of *Trichomonas *in men, particularly when the diagnosis is based solely on the cytological evaluation of poorly preserved organisms in a urine specimen. The diagnosis may be especially problematic when the patient denies sexual contact, or when a sexually transmitted disease in a particular patient, such as an infant [7] or the elderly, seems implausible. Adding to the problem is the fact that when in urine, trichomonads usually assume variable shapes [8]. Parasite variation in size and shape may be further exaggerated in air-dried urine smears [9]. In male patients, the organisms also tend to be somewhat smaller than their counterpart in female patients [10]. In addition to the present patient, our laboratory has diagnosed *Trichomonas *in Papanicolaou-stained urine specimens in eight men of mean age 59 years (range 47–82 years), over a 15 year period. This finding is in keeping with published data suggesting that higher organism loads occur in older men [11]. We do not know if any of these individuals also had diabetes.

In conclusion, as illustrated in the case presented, we recommend the use of p16 immunocytochemistry to help establish the diagnosis of *Trichomonas *in unexpected patients or specimen types in which potential trichomonads are identified following routine cytologic evaluation.

## References

[B1] Aslan DL, McKeon DM, Stelow EB, Gulbahce HE, Kjeldahl K, Pambuccian SE (2005). The diagnosis of trichomonas vaginalis in liquid-based Pap tests: Morphological characteristics. Diagn Cytopathol.

[B2] Aslan DL, Gulbahce HE, Stelow EB, Setty S, Brown CA, McGlennen RC, Pambuccian SE (2005). The diagnosis of Trichomonas vaginalis in liquid-based Pap tests: Correlation with PCR. Diagn Cytopathol.

[B3] Pantanowitz L, Florence RR, Goulart RA, Otis CN (2005). *Trichomonas vaginalis *p16 immunoreactivity in cervicovaginal pap tests: a diagnostic pitfall. Modern Pathology.

[B4] Bibbo M, Klump WJ, DeCecco J, Kovatich AJ (2002). Procedure for immunocytochemical detection of P16^INK4A ^antigen in thin-layer, liquid-based specimens. Acta Cytol.

[B5] Saqi A, Pasha TL, McGrath CM, Yu GH, Zhang P, Gupta P (2002). Overexpression of p16^INK4A ^in liquid-based specimens (SurePath™) as marker of cervical dysplasia and neoplasia. Diagn Cytopathol.

[B6] Sahebali S, Depuydt CE, Segers K, Moeneclaey LM, Vereecken AJ, Van Marck E, Bogers JJ (2004). P16^INK4a ^as an adjunct marker in liquid-based cervical cytology. Int J Cancer.

[B7] Schares T, Machtinger S, D'Harlingue AE, Maloney JR (1982). *Trichomonas vaginalis *urinary tract infection in an infant. Pediatr Infect Dis.

[B8] Malyszko E, Januszko T (1991). Detection of *Trichomonas vaginalis *in men [Article in Polish]. Pol Tyg Lek.

[B9] Loo CK, Gune S (2000). *Trichomonas vaginalis *in urine cytology. Acta Cytol.

[B10] Summers JL, Ford ML (1972). The Papanicolaou smear as a diagnostic tool in male trichomoniasis. J Urol.

[B11] Wendel KA, Erbelding EJ, Gaydos CA, Rompalo AM (2003). Use of urine polymerase chain reaction to define the prevalence and clinical presentation of *Trichomonas vaginalis *in men attending an STD clinic. Sex Transm Infect.

